# Real-Time Indoor Visible Light Positioning (VLP) Using Long Short Term Memory Neural Network (LSTM-NN) with Principal Component Analysis (PCA)

**DOI:** 10.3390/s24165424

**Published:** 2024-08-22

**Authors:** Yueh-Han Shu, Yun-Han Chang, Yuan-Zeng Lin, Chi-Wai Chow

**Affiliations:** Department of Photonics & Graduate Institute of Electro-Optical Engineering, College of Electrical and Computer Engineering, National Yang Ming Chiao Tung University, Hsinchu 30010, Taiwan

**Keywords:** visible light communication (VLC), visible light positioning (VLP), long short-term memory neural network (LSTM-NN), principal component analysis (PCA)

## Abstract

New applications such as augmented reality/virtual reality (AR/VR), Internet-of-Things (IOT), autonomous mobile robot (AMR) services, etc., require high reliability and high accuracy real-time positioning and tracking of persons and devices in indoor areas. Among the different visible-light-positioning (VLP) schemes, such as proximity, time-of-arrival (TOA), time-difference-of-arrival (TDOA), angle-of-arrival (AOA), and received-signal-strength (RSS), the RSS scheme is relatively easy to implement. Among these VLP methods, the RSS method is simple and efficient. As the received optical power has an inverse relationship with the distance between the LED transmitter (Tx) and the photodiode (PD) receiver (Rx), position information can be estimated by studying the received optical power from different Txs. In this work, we propose and experimentally demonstrate a real-time VLP system utilizing long short-term memory neural network (LSTM-NN) with principal component analysis (PCA) to mitigate high positioning error, particularly at the positioning unit cell boundaries. Experimental results show that in a positioning unit cell of 100 × 100 × 250 cm^3^, the average positioning error is 5.912 cm when using LSTM-NN only. By utilizing the PCA, we can observe that the positioning accuracy can be significantly enhanced to 1.806 cm, particularly at the unit cell boundaries and cell corners, showing a positioning error reduction of 69.45%. In the cumulative distribution function (CDF) measurements, when using only the LSTM-NN model, the positioning error of 95% of the experimental data is >15 cm; while using the LSTM-NN with PCA model, the error is reduced to <5 cm. In addition, we also experimentally demonstrate that the proposed real-time VLP system can also be used to predict the direction and the trajectory of the moving Rx.

## 1. Introduction

Currently, the growing demand of applications such as augmented reality/virtual reality (AR/VR), Internet-of-Things (IOT), autonomous mobile robot (AMR) services, etc., require high precision positioning in indoor environments. In addition, in many indoor and multiple-level facilities, people often have difficulties in finding their destinations. In some places, such as factories and warehouses, where robots have to move around or to collaborate with humans; reliable, high-accuracy and real-time indoor positioning systems (IPS) are crucial. Nowadays, Global Positioning System (GPS) can provide high accuracy positioning for outdoor environments. However, as GPS needs to obtain positioning information from satellites, it cannot provide reliable and accurate positioning for indoor and underground environments. Radio-frequency (RF) based IPS, such as Bluetooth, Wireless Fidelity (WiFi), and wireless local area networks (WLANs) have attracted much attention [1,2]; however, their implementations still face many challenges, such as electromagnetic interference (EMI) with nearby RF devices or limited IPS accuracy [3,4].

Over the past decades, visible light communication (VLC) has gained increasing attention. Many reliable [5,6,7,8,9,10,11,12] and high data rate VLC transmissions [13,14,15,16,17,18,19] have been reported. Apart from providing data transmission, these VLC systems can also provide high accuracy indoor visible light positioning (VLP) [20,21]. In addition, predicting the position using VLP is also useful for parameter optimization in VLC systems [22]. VLP can be implemented based on the existing indoor light emitting diode (LED) illumination system; hence, it can be cost-effective and energy-effective. Different VLP systems have been realized, and their positioning mechanisms including proximity [23], time-of-arrival (TOA) [24], time-difference-of-arrival (TDOA) [25], angle-of-arrival (AOA) [26,27], and received-signal-strength (RSS) [28,29]. In order to enhance the positioning accuracy and reduce the influence of ambient lights and reflections by surrounding objects, artificial intelligence/machine learning (AI/ML) have also been proposed, including regression [30,31,32], kernel ridge regression [33], artificial neural network (ANN) [34], long short-term memory neural network (LSTM-NN) [27], and convolutional neural network (CNN) [35].

Among these VLP methods, the RSS method is simple and efficient. As the received optical power has an inverse relationship with the distance between the LED transmitter (Tx) and the photodiode (PD) receiver (Rx), position information can be estimated by studying the received optical power from different Txs. The RSS positioning scheme can be classified into server-based and client-based architectures depending on which side is responsible for the positioning estimation [36]. In the server-based architecture, the user equipment (UE) or mobile device reports its RSS information to the server and requests its positioning information. In the client-based architecture, the UE or mobile device predicts its location based on different RSS received from surrounding access points (APs). Although the client-based architecture requires a higher processing power in the UE, the user privacy can be protected. In the literature, most RSS-based VLP systems are processed off-line with stationary Rxs. For real-time VLP systems in which the Rxs could be moving continuously, the received RSS data will also vary continuously over time at different locations. An efficient AI/ML model, which can take into account time varying information, is needed. Here, the LSTM-NN model is utilized.

In this work, we propose and experimentally demonstrate a real-time VLP system utilizing LSTM-NN with principal component analysis (PCA) to mitigate the high positioning error particularly at the positioning unit cell boundaries. PAC is a dimensionality reduction scheme by transforming features to a lower dimension space. It can select and emphasize the most informative features in the VLP system (e.g., RSS signal with weak signal-to-noise ratio, SNR), while de-emphasizing the last informative features (e.g., noises and reflections). As a result, the positioning accuracy can be enhanced. Additionally, we also illustrate real-time VLP positioning. We believe that the proposed work here has improved scalability and robustness compared with other works. Here, we employ client-based positioning architecture, which can protect user privacy. Experimental results show that in a positioning unit cell of 100 × 100 × 250 cm^3^, the average positioning error is 5.912 cm when using LSTM-NN only. By utilizing the PCA, we can observe that the positioning accuracy can be significantly enhanced to 1.806 cm, showing a positioning error reduction of 69.45%. In addition, we also demonstrate experimentally that the proposed real-time VLP system can also be used to predict the direction and the trajectory of the moving Rx.

## 2. Algorithm and Experiment

**Figure 1:**
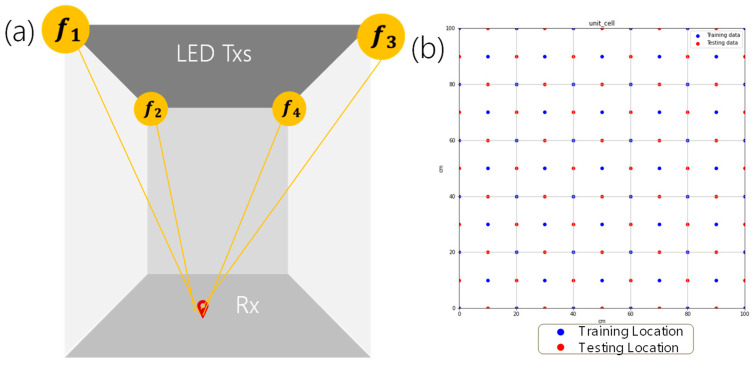
(**a**) Architecture of the VLP system with four LEDs modulated by specific RF carrier frequencies of *f*_1_, *f*_2_, *f*_3_, and *f*_4_, (47 kHz, 59 kHz, 83 kHz, 101 kHz), respectively. (**b**) Bird-view of the positioning unit cell indicating the training and testing locations.

The architecture of the VLP system is shown in Figure 1a. The positioning unit cell consists of four white LED lamps, which are commercially available. Each LED lamp has an output power of 13 W. Each LED lamp is electrically driven by a direct-current (DC) voltage and RF signal combined via a bias-tee. RF carrier frequencies at *f*_1_ = 47 kHz, *f*_2_ = 59 kHz, *f*_3_ = 83 kHz, and *f*_4_ = 101 kHz are used to modulate the four LEDs, respectively, as shown in Figure 1a. The selected RF carrier frequencies should be less than the direct modulation frequency of the white-light LED lamp, which is ~ MHz. As fast Fourier transform (FFT) will be used in the VLP RSS decoding processing, in order to provide accurate VLP prediction, the selected RF carrier frequencies should be well separated among themselves. In addition, the use of odd frequencies can avoid harmonic frequency overlapping during the RSS detection after the FFT. Figure 1b illustrates the bird-view of the positioning unit cell indicating the training and testing locations. The ground truth locations are marked on the floor. There are 61 training and 60 testing locations. During the training and testing phases, each location is measured 20 times. The positioning unit cell has dimensions of 100 × 100 cm^2^ as shown in Figure 1b. The distance between the LED Tx plane and the PD is 250 cm. As shown in Figure 1b, the training and testing locations are on grids. During the experiment, we manually marked the ground truth coordinates on the floor using rulers. Here, the training and testing points on rectangular grids are easier to be marked manually. We believe that as long as the training data are sufficient, similar performance can be obtained if the testing locations are randomly selected outside the grid points. It is worth noting that the VLP performance could be affected by factors, such as the emitted power and the number of LEDs in the positioning unit cell. Increasing the power of LEDs could increase the SNR received by the PD and increase the VLP performance; however, in this demonstration, we use commercially available, white-LED lamps, and they provide fixed output powers for typical indoor illumination. Additionally, increasing the number of LEDs in the positioning unit cell could increase the VLP performance; however, it may increase the system complexity.

**Figure 2:**
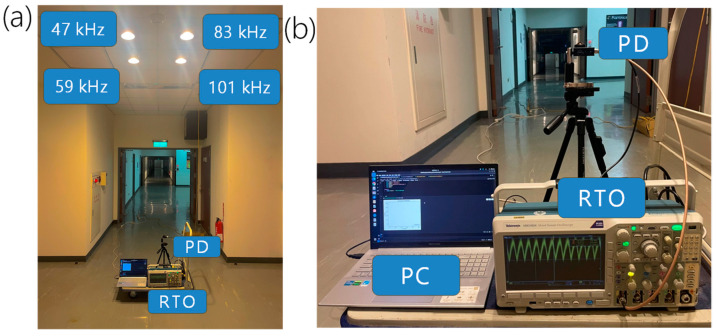
(**a**) Experimental photo of the VLP experiment. (**b**) Photo of the client side. The PD, RTO, and PC are all placed on a trolley for training and testing data collections. PD: photodiode; RTO: real-time oscilloscope.

Figure 2a shows the experimental photo of the VLP experiment. The proof-of-concept experiment was performed on the 2/F at the corridor outside Rm 201 laboratory at the Tin Ka Ping building, National Yang Ming Chiao Tung University. As shown in Figure 2a, four commercially available white LED lamps were installed on the ceiling to provide both lighting and VLP. Figure 2b is the photo of the client side. A PD is connected to a real-time-oscilloscope (RTO), which is then attached to a laptop personal computer (PC) to collect and analyze RSS data. The RTO acts as the analog-to-digital converter (ADC) to digitize the received optical signals obtained from the PD for real-time positioning analysis. Other ADC devices or circuits that have enough signal bandwidth and can process four channels simultaneously can be used. The use of RTO here is because other ADC devices were not available in the laboratory during the experiment. The PD is mounted on a tripod with a bubble level balance tool to ensure the horizontal position of the PD. The PD, RTO, and PC are all placed on a trolley for training and testing data collections. The separation between the Rx plane and the LED plane is 250 cm; hence, the VLP positioning unit cell has dimensions of about 100 × 100 × 250 cm^3^. Here, the dimensions of the positioning unit cell are restricted by our experimental environment. As long as the whole positioning unit cell can be covered by the lights emitted by all the LEDs, high accuracy can be achieved even if the unit cell has different dimensions.

**Figure 3:**
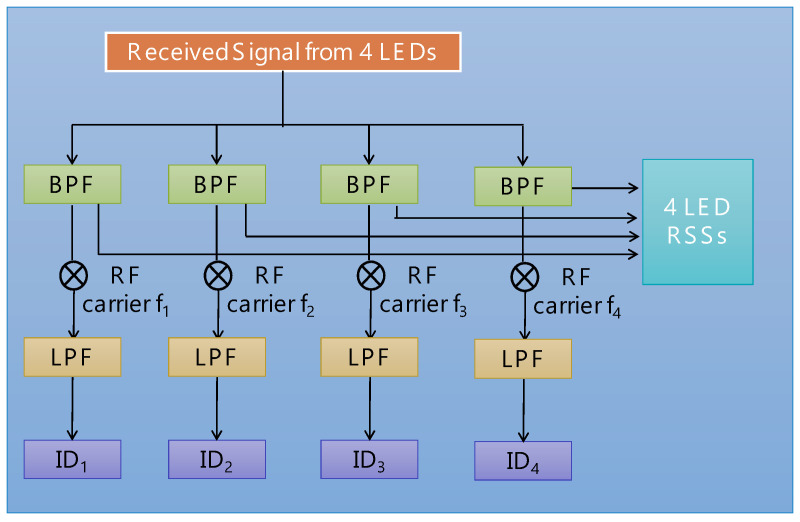
Architecture of the VLP Rx. ID: optical identifier; BPF: band-pass filter; LPF: low-pass filter.

Figure 3 shows the architecture of the VLP Rx. A PD obtains the visible signals from four LEDs simultaneously for the VLP. The RTO then performs the ADC. Then, the optical identifiers (IDs) and RSS signals are obtained. The four optical IDs can be used to identify on which unit cell the client Rx is positioned, while the four RSS values can be used to locate the precise coordinates of the client Rx inside that particular unit cell. Specific frequency band-pass filters (BPFs) at of 47 kHz, 59 kHz, 83 kHz, and 101 kHz inside the Rx architecture can be used to filter and select the RSS values. Then, each signal band is down-converted, and the optical ID is obtained via a low-pass filter (LPF). At each location, the PD can receive four RSS data ($p_{1}$, $p_{2}$, $p_{3}$, $p_{4}$). In order to enhance the machine learning model performance and explore the relationship among these features, feature expansion based on the multiplication among the RSS data is performed. Here, in order to reduce the complexity and the processing time, up to the second order term is used. Hence, 15 features including the first and second order after feature extraction are obtained (1, $p_{1}$, $p_{2}$, $p_{3}$, $p_{4}$, $p_{1}^{2},\;p_{1}p_{2},\;p_{1}p_{3},\;p_{1}p_{4}$, $p_{2}^{2}$, $p_{2}p_{3},\;p_{2}p_{4}$, $p_{3}^{2},\;p_{3}p_{4},$ $p_{4}^{2}$). They are employed as the features of the LSTM-NN model, and the corresponding coordinates (*x*, *y*) are utilized as the model labels. For a larger environment, the whole area can be divided into several smaller positioning unit cells, and each consists of four LED light sources. As shown in Figure 1a, each LED will transmit its ID information in an on–off keying (OOK) format carried by the RF carriers. After demodulating the four IDs from the four LEDs as illustrated in Figure 3, the positioning unit cell where the client Rx locates at can be known. When the client moves to another unit cell served by different LEDs, new IDs will be updated, so that the new positioning unit cell can be determined.

As shown in Figure 3, the RSS data obtained from the four LEDs will be filtered by different band-pass filters (BPFs) with their center frequencies matching with the corresponding RF carrier frequencies emitted by the four LEDs. The interference from the ambient light source could be reduced. In addition, The PD and LED used in the proposed VLP system are commercially available. There is no special requirement for them. As different LEDs from different brands may have different emission profiles, to achieve high accuracy positioning, the LEDs used in the model training phase and testing phase should be the same. If the LEDs are replaced later, the model should be re-trained.

**Figure 4:**
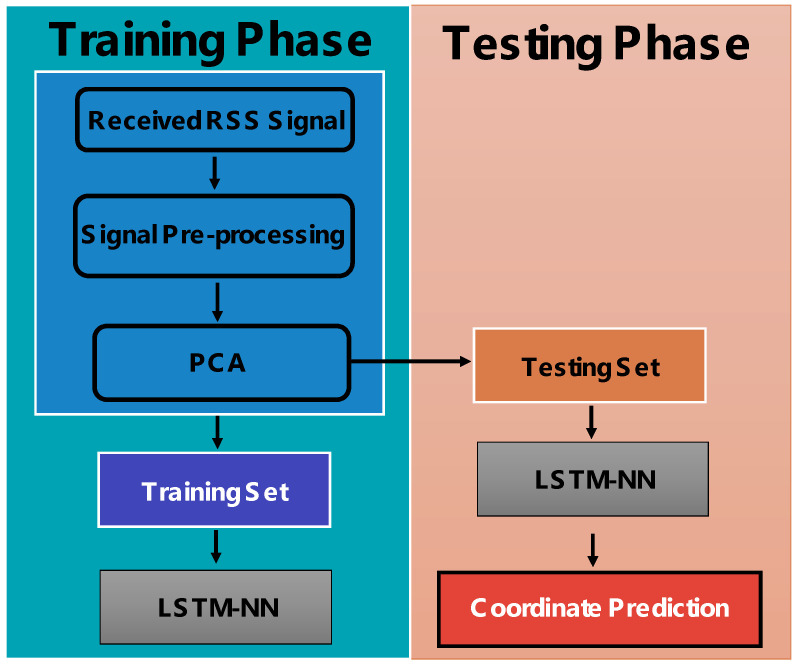
Flow diagram of the proposed real-time VLP system utilizing LSTM-NN with PCA.

Figure 4 shows the flow diagram of the proposed real-time VLP system utilizing LSTM-NN with PCA to mitigate the high positioning error. There are two phases: the training phase and testing phase. After the four RSS values are obtained by the ADC, signal pre-processing is performed. In the signal pre-processing module, the four RSS data ($p_{1}$, $p_{2}$, $p_{3}$, $p_{4}$) at each location will be extended with a cross-term to 15 data to increase the performance of feature extraction (1, $p_{1}$, $p_{2}$, $p_{3}$, $p_{4}$, $p_{1}^{2},\;p_{1}p_{2},\;p_{1}p_{3},\;p_{1}p_{4}$, $p_{2}^{2}$, $p_{2}p_{3},\;p_{2}p_{4}$, $p_{3}^{2},\;p_{3}p_{4},$ $p_{4}^{2}$). Then the data will be proceeded by PCA and separated into the training data set and testing data set according to their locations as shown in Figure 4.

**Figure 5:**
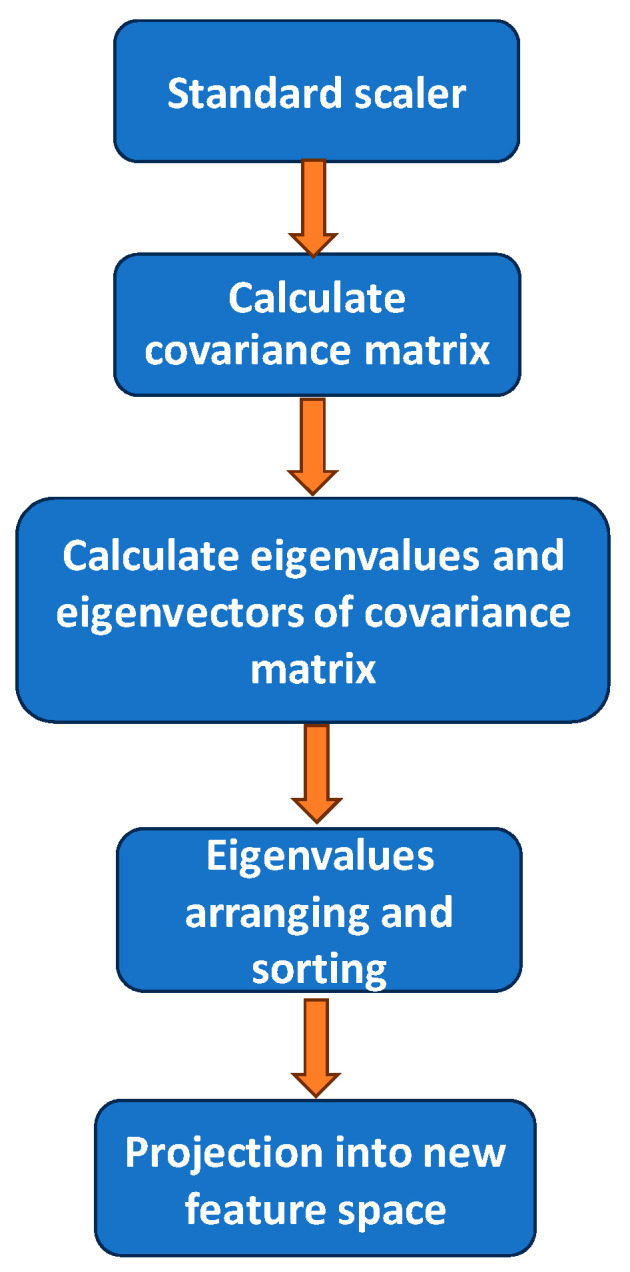
Flow diagram of the PCA used in the VLP experiment.

PAC is a dimensionality reduction scheme by transforming features to a lower dimension space [36,37]. It utilizes a covariance matrix to decorrelate the features and to data project in the direction of the most significant variance. Hence, PCA can be applied to select and emphasize the most informative features in the VLP system, while de-emphasizing the last informative features, such as noises and reflections. As a result, the positioning accuracy can be enhanced. Figure 5 shows the flow diagram of the PCA used in the VLP experiment; it includes several processes, such as, performing standard-scaler, covariance matrix calculation, eigenvalue and eigenvector calculation based on the covariance matrix, eigenvalue arranging and sorting, and finally projection into new feature space.

The first step in the PCA is performing standard-scaler, which is the Z-score normalization used in statistics. Equation (1) shows the equation of Z-score normalization, where $p_{i}$ is the RSS value of the *i*-th LED at one position point; $\mu_{i}$ and $\sigma_{i}$ are the mean and the standard deviation of the *i*-th LED:

(1) $$z=\frac{\left(p_{i}-\mu_{i}\right)}{\sigma_{i}}$$

The second step is to calculate the covariance matrix **C**. Assuming a data set **X** having *N* samples and *p* features, the covariance matrix **C** can be expressed as Equation (2):

(2) $$C=\overset{N}{\underset{k=1}{\sum }}\left(X_{k}-\bar{X}\right){\left(X_{k}-\bar{X}\right)}^{T}$$

and $\bar{X}$ is the sample mean, as shown in Equation (3):

(3) $$\bar{X}=\overset{N}{\underset{k=1}{\sum }}\frac{X}{N}$$

Then, it is to let the eigenvalues of **C** $\lambda_{i}=\{\begin{array}{ccc} \lambda_{1}, & \ldots , & \lambda_{D} \end{array}\}$ be arranged in descending order with the corresponding eigenvector $\nu_{i}=\{\begin{array}{ccc} \nu_{1}, & \ldots , & \nu_{D} \end{array}\}$, so that they can satisfy Equation (4):

(4) $$C\nu_{i}=\lambda_{i}\nu_{i}$$

The eigenvector here represents the main direction of the data, and the corresponding eigenvalue represents the amount of data variability in that direction. Then, the eigenvalues should be arranged and sorted in descending order according to the size, $\begin{array}{ccc} \lambda_{1}\geq \lambda_{2}\geq & \ldots \geq & \lambda_{D} \end{array}$ and the top k eigenvalues selected. These k eigenvalues will constitute a new feature space (i.e., principal component space). Selecting the minimum number of eigenvalues can remove duplication and reduce the noise of data. In this work, the first 15 eigenvalues are kept for the projection into feature space. This can be mathematically represented in Equation (5):

(5) $$X_{NEW}=X\nu_{i}$$

**Figure 6:**
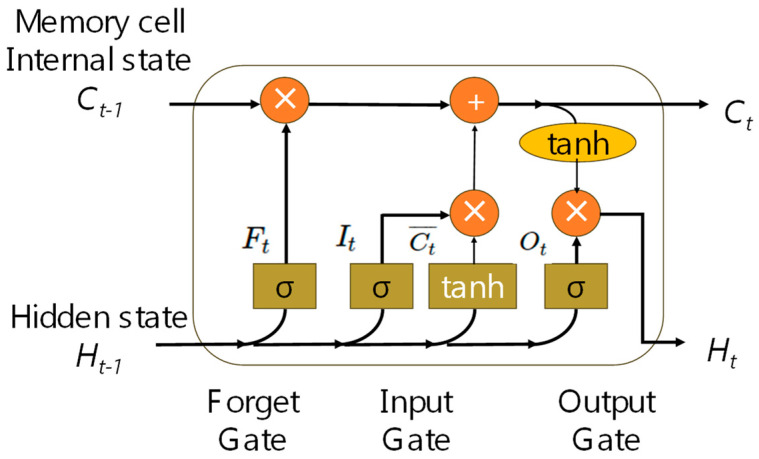
Structure of an LSTM cell used in the LSTM-NN model.

After the PCA, the data will pass to the LSTM-NN model for VLP prediction. LSTM-NN can mitigate the signal fluctuations using its temporal memory characteristics. Figure 6 shows the structure of an LSTM cell used in the LSTM-NN model. Each LSTM cell consists of three important control gates as follows: input gate *I*, forget gate *F*, and output gate *O*. Each of these three gates has a sigmoid function to control the output values between 0 and 1. They can be mathematically expressed in Equation (6):

(6) $$I_{t}=\sigma \left(W_{i}\left[H_{t-1},\;X_{t}\right]+b_{i}\right)\;F_{t}=\sigma \left(W_{f}\left[H_{t-1},\;X_{t}\right]+b_{f}\right)\;O_{t}=\sigma \left(W_{o}\left[H_{t-1},\;X_{t}\right]+b_{o}\right)$$

where *W* is weight matrix and *b* is the bias. Inside the LSTM cell, the candidate memory unit is the difference from the previous three gates. It uses a different activation function tanh to produce the output values between –1 and 1, and it can be mathematically expressed in Equation (7):

(7) $$\bar{C_{t}}=\tan h\left(W_{c}\left[H_{t-1},\;X_{t}\right]+b_{C}\right)$$

The memory unit can also act as a mechanism to control input and forget, as expressed in Equation (8):

(8) $$C_{t}=F_{t}\times C_{t-1}+I_{t}\times \bar{C_{t}}$$

Equation (9) shows the hidden state. When the output gate is close to 1, it can effectively pass all the memory information to the prediction part. When the output gate is close to 0, it only retains all the information in the memory cell without updating the hidden state:

(9) $$H_{t}=O_{t}\times \tan h(C_{t-1})$$

**Figure 7:**
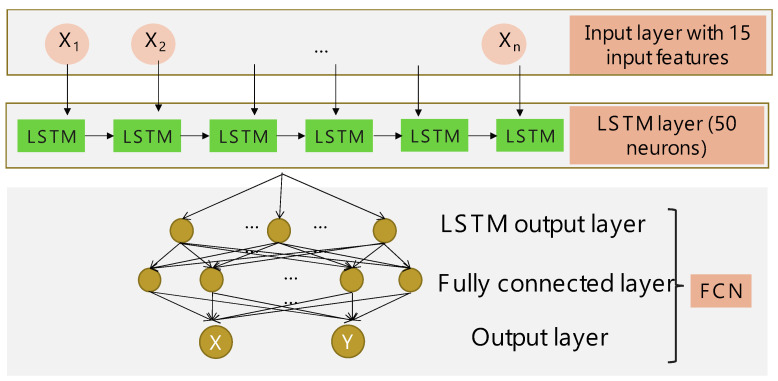
Structure of the proposed LSTM-NN model used in both training phase and testing phase.

Figure 7 shows the structure of the proposed LSTM-NN model used in both the training phase and testing phase. The input layer receives the 15 features obtained from the feature extraction (1, $p_{1}$, $p_{2}$, $p_{3}$, $p_{4}$, $p_{1}^{2},\;p_{1}p_{2},\;p_{1}p_{3},\;p_{1}p_{4}$, $p_{2}^{2}$, $p_{2}p_{3},\;p_{2}p_{4}$, $p_{3}^{2},\;p_{3}p_{4},$ $p_{4}^{2}$). The LSTM-NN has a single LSTM layer with neuron number of 50, and the activation function is ReLU. The last three layers are a fully-connected network (FCN). The loss function is the mean square error (MSE) and the optimizer is Adam for parameter update during the training phase. It takes about 200 epochs for the model to fully converge.

## 3. Results and Discussion

**Figure 8:**
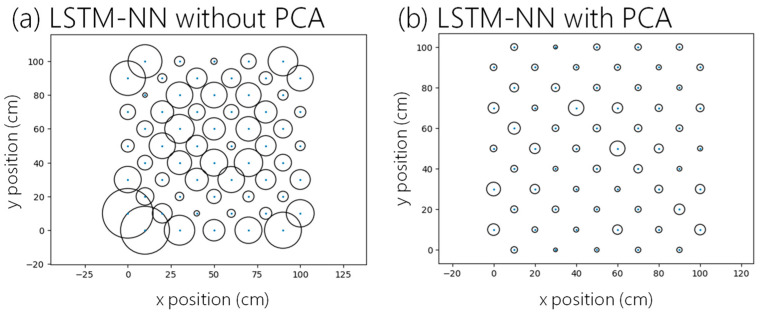
Error distributions using (**a**) the LSTM-NN only and (**b**) the LSTM-NN with PCA.

Figure 8a,b shows the testing data average error distributions using the LSTM-NN only and using the LSTM-NN with PCA. The blue dots and the radii of circles are the testing location and the average error, respectively. The average positioning error can be obtained by using the root mean square error as shown in Equation (10), where *N* is the number of measurements, *X* and *Y* are the x- and y-coordinates, respectively, prediction and label are the coordinates obtained from the models and actual coordinates, respectively:

(10) $$average\;error=\sqrt{\frac{\sum_{N}{\left(X_{prediction}-X_{label}\right)}^{2}+{\left(Y_{prediction}-Y_{label}\right)}^{2}}{N}}$$

We can observe that the proposed LSTM-NN with PCA can significantly improve the VLP error, as illustrated by the smaller circles in Figure 8b. As illustrated in Figure 8a, higher positioning errors occur at the four corners of the positioning unit cell. This is because when the PD is located at the corner, the received optical signal emitted by the LED lamp from the opposite corner is weak. For example, if the PD locates at the left bottom corner, it will receive a weak signal from the LED at the right top corner. The lower SNR will increase the Rx noise and reduce the positioning accuracy. In addition, the positioning unit cell boundaries suffer from the reflection noise caused by the walls. The proposed PCA can select and emphasize the most informative features in the VLP system (e.g., RSS from the opposite corner LED), while de-emphasizing the last informative features (e.g., noises and reflections). As a result, the positioning accuracy can be enhanced. In a positioning unit cell of 100 × 100 cm^2^, the average positioning error is 5.912 cm when using LSTM-NN only. By utilizing the LSTM-NN with PCA, the positioning accuracy can be significantly enhanced to 1.806 cm, indicating a positioning error reduction of 69.45%. The experimental result shows that the LSTM-NN with PCA can mitigate the positioning error effectively.

**Figure 9:**
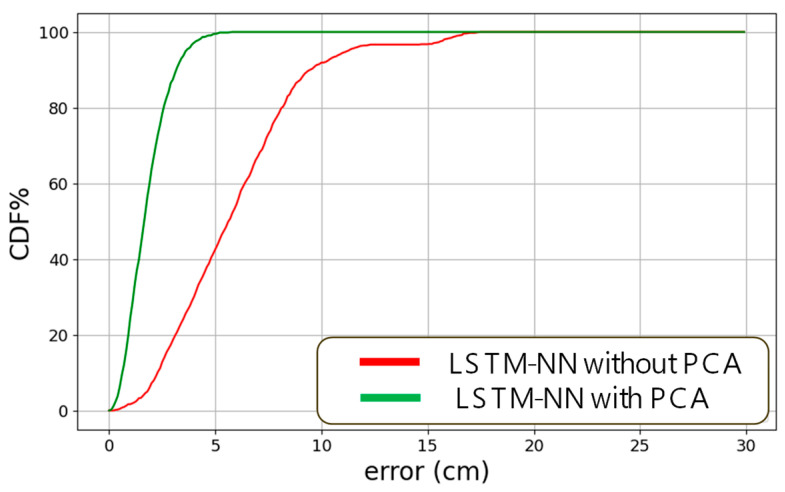
CDF of the measured positioning error using LSTM-NN only and using the LSTM-NN with PCA.

Figure 9 shows the cumulative distribution function (CDF) of the measured positioning error using LSTM-NN only and using the LSTM-NN with PCA, respectively. Without PCA, the positioning error of 80% of the experimental data is within 8.5 cm; while using the PCA, the errors are within 2.5 cm. Without PCA, the positioning error of 95% of the experimental data is within 12 cm; while using the LSTM-NN with the PCA model, the error is reduced to within 3.6 cm.

**Figure 10:**
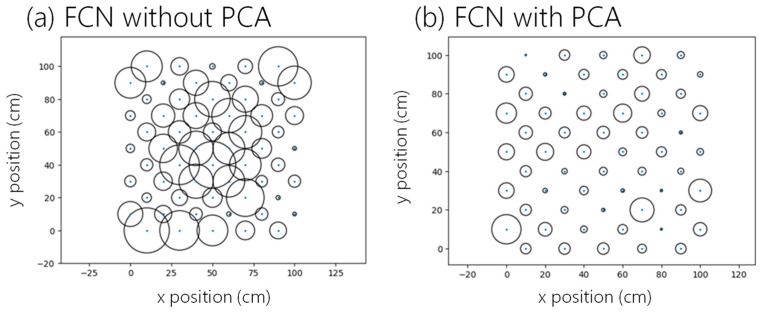
Error distributions using (**a**) FCN only and (**b**) FCN with PCA.

**Figure 11:**
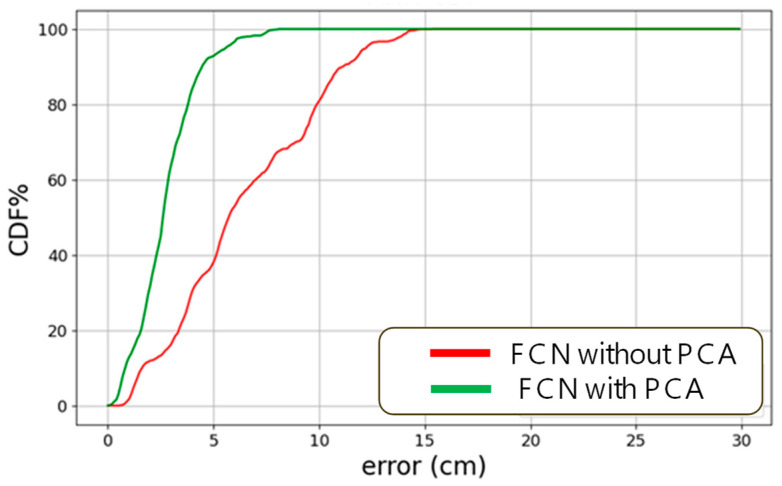
CDF of the measured positioning error using FCN only and using FCN with PCA.

Here, we also compare the proposed LSTM-NN with the fully connected neural network (FCN) without the LSTM layers shown in Figure 7. Figure 10a,b shows the testing data average error distributions using the FCN only and using the FCN with PCA. The average positioning errors when using FCN only and FCN with PCA are 6.414 cm and 2.722 cm, respectively. When comparing Figure 8a and Figure 10a, we can observe that the LSTM layers can significantly reduce the positioning error. In addition, when comparing Figure 8b and Figure 10b with the application of PCA, we can observe that PCA is effective in both schemes; and LSTM-NN with PCA outperforms the others. Figure 11 shows the CDF of the measured positioning error using FCN only and using the FCN with PCA, respectively. Without PCA, the positioning error of 80% of the experimental data is within 10 cm; while using the PCA, the errors are within 3.8 cm. Without PCA, the positioning error of 95% of the experimental data is within 12.2 cm; while using FCN with the PCA model, the error is reduced to within 5.6 cm.

**Figure 12:**
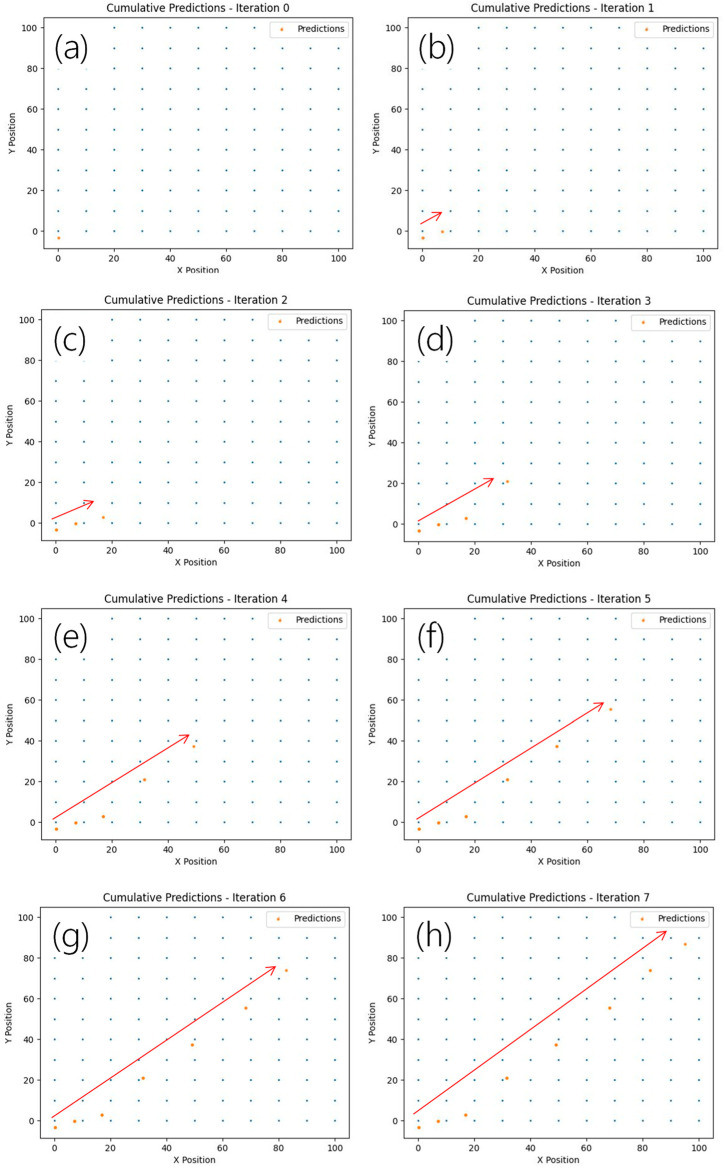
Experimental predicted location of the moving Rx using the LSTM-NN with PCA at different iterations. (**a**–**h**) Indication of predicted direction and trajectory of the Rx from iteration 1 to 7.

The proposed real-time VLP system can also be used to predict the direction and the trajectory of the moving Rx. As shown in Figure 2b, the PD, RTO and laptop PC are all placed on a trolley for the trajectory prediction. The trolley starts from the coordinate (0, 0) and moves to the coordinate (100, 100). Figure 12a–h shows the experimental predicted location of the moving Rx using the LSTM-NN with PCA at different iterations. Here, the red arrow indicates the moving trace of the Rx. The blue and orange dots indicate the coordinates of the unit cell and the predicated location of the moving Rx in different iterations. It is worth mentioning that the training data are obtained from stationary Rx; hence, the positioning error of the moving Rx is slightly higher. The time for each iteration is <1 s. It can be observed that even within a small unit cell of 100 × 100 × 250 cm^3^, the predicted direction and trajectory of the Rx match the actual Rx movement.

One potential application scenario of this work is that it can locate robots in indoor environments. With the help of the positioning, these robots can deliver goods in warehouses or medicine to patients in hospitals efficiently. Additionally, another potential application scenario is that it can identify the locations of wheelchairs in hospitals or trolleys in supermarkets efficiently. As discuss above, RF based indoor positioning systems, such as Bluetooth, WiFi, etc., can also offer high accuracy positioning. However, their implementation could be limited by interferences caused by different RF devices nearby. According to the survey in [38], the state-of-the-art RF-assisted indoor positioning systems could provide within meter to sub-meter accuracy. We believe that our proposed work with accuracy within centimeters could be a competitive indoor positioning solution.

It is worth noting that in the proposed scheme, the positioning accuracy will be reduced in more complex indoor environments with obstacles. The LSTM-NN model should be retrained when obstacles present. Previous work suggested using a lighting design tool, such as DIALux, to simulate the indoor environment with obstacles [39]; and the simulated results could reduce the machine learning training burden for the VLP system. It is also worth mentioning that as long as the whole positioning unit cell can be covered by the lights emitted by all the LEDs in that positioning unit cell, high accuracy can be achieved even if the unit cell has different shapes. In this proof-of-concept demonstration, it takes about 1 s for our RTO to capture the RSS data from the PD and for the PC to execute the LSTM-NN model for positioning prediction. We believe that this process can be speeded up by using hardware decoding, such as using field programmable gate array (FPGA) board. As the processing latency is about 1 s, the criteria for each iteration reported in Figure 12a,h should be more than 1 s, allowing it to have enough time for the VLP prediction.

## 4. Conclusions

New applications and services require high reliability and high accuracy real-time positioning and tracking of persons and devices in indoor areas—such as identifying the locations of wheelchairs in hospitals or trolleys in supermarkets. In this work, we proposed and demonstrated experimentally a real-time VLP system utilizing LSTM-NN with PCA to mitigate the high positioning error particularly at the positioning unit cell boundaries. For the input to the LSTM-NN model, four RSS data ($p_{1}$, $p_{2}$, $p_{3}$, $p_{4}$) at each location were extended with cross-term to 15 data to increase the performance of feature extraction (1, $p_{1}$, $p_{2}$, $p_{3}$, $p_{4}$, $p_{1}^{2},\;p_{1}p_{2},\;p_{1}p_{3},\;p_{1}p_{4}$, $p_{2}^{2}$, $p_{2}p_{3},\;p_{2}p_{4}$, $p_{3}^{2},\;p_{3}p_{4},$ $p_{4}^{2}$). They were employed as the features of the LSTM-NN model, and the corresponding coordinates (*x*, *y*) were utilized as the labels of the model. The PAC is a dimensionality reduction scheme by transforming features to a lower dimension space. It utilizes covariance matrix to decorrelate the features and to data project in the direction of the most significant variance. Hence, PCA can be applied to select and emphasize the most informative features in the VLP system, while de-emphasizing the last informative features, such as noises and reflections. As a result, the positioning accuracy can be enhanced. Experimental results show that in a positioning unit cell of 100 × 100 cm^2^, the average positioning error is 5.912 cm when using LSTM-NN only. By utilizing the LSTM-NN with PCA, the positioning accuracy can be significantly enhanced to 1.806 cm, indicating a positioning error reduction of 69.45%. This significant improvement of positioning accuracy to within 2 cm could facilitate the effective implementation of high precision applications, such as AR/VR, IOT, AMR services, using visible light. In the CDF measurements, without PCA, the positioning error of 80% of the experimental data is within 8.5 cm; while using PCA, the errors are within 2.5 cm. Without PCA, the positioning error of 95% of the experimental data is within 12 cm; while using the LSTM-NN with the PCA model, the error is reduced to within 3.6 cm. In addition, we also demonstrate experimentally that the proposed real-time VLP system can also be used to predict the direction and the trajectory of the moving Rx. In this proof-of-concept demonstration, it takes about 1 s for our RTO to capture the RSS data from the PD and for the PC to execute the LSTM-NN model for positioning prediction. We believe that this process can be speeded up by using hardware decoding, such as using FPGA board. Possible future explorations could involve testing in larger or more complex environments, integrating different types of sensors, or evaluating dynamic environments, such as moving obstacles or people.

## Data Availability

The data presented in this study are available on request from the corresponding author.

## References

[1] Yang C., Shao H. (2015). WiFi-based indoor positioning. IEEE Commun. Mag..

[2] Faragher R., Harle R. (2015). Location fingerprinting with Bluetooth low energy beacons. IEEE J. Sel. Areas Commun..

[3] Tran H.Q., Ha C. (2022). Machine learning in indoor visible light positioning systems: A review. Neurocomputing.

[4] Rahman A.B.M.M., Li T., Wang Y. (2020). Recent advances in indoor localization via visible lights: A survey. Sensors.

[5] Haas H., Elmirghani J., White I. (2020). Optical wireless communication. Phil. Trans. R. Soc..

[6] Komine T., Nakagawa M. (2004). Fundamental analysis for visible-light communication system using LED lights. IEEE Trans. Con. Electron..

[7] O’Brien D.C., Zeng L., Le-Minh H., Faulkner G., Walewski J.W., Randel S. Visible light communications: Challenges and possibilities. Proceedings of the 2008 IEEE 19th International Symposium on Personal, Indoor and Mobile Radio Communications.

[8] Chow C.W., Yeh C.H., Liu Y.F., Liu Y. (2011). Improved modulation speed of LED visible light communication system integrated to main electricity network. Electron. Lett..

[9] Yu T.C., Huang W.T., Lee W.B., Chow C.W., Chang S.W., Kuo H.C. (2021). Visible light communication system technology review: Devices, architectures, and applications. Crystals.

[10] Chow C.W. (2024). Recent advances and future perspectives in optical wireless communication, free space optical communication and sensing for 6G. J. Lightwave Technol..

[11] Chi N., Zhou Y., Wei Y., Hu F. (2020). Visible light communication in 6G: Advances, challenges, and prospects. IEEE Vehicular Technol. Mag..

[12] Chow C.W., Liu Y., Yeh C.H., Chang Y.H., Lin Y.S., Hsu K.L., Liao X.L., Lin K.H. (2021). Display light panel and rolling shutter image sensor based optical camera communication (OCC) using frame-averaging background removal and neural network. J. Lightwave Technol..

[13] Cossu G., Khalid A.M., Choudhury P., Corsini R., Ciaramella E. (2012). 3.4 Gbit/s visible optical wireless transmission based on RGB LED. Opt. Exp..

[14] Zhu X., Wang F., Shi M., Chi N., Liu J., Jiang F. 10.72Gb/s visible light communication system based on single packaged RGBYC LED utilizing QAM-DMT modulation with hardware pre-equalization. Proceedings of the 2018 Optical Fiber Communications Conference and Exposition (OFC).

[15] Chi Y.C., Hsieh D.H., Tsai C.T., Chen H.Y., Kuo H.C., Lin G.R. (2015). 450-nm GaN laser diode enables high-speed visible light communication with 9-Gbps QAM-OFDM. Opt. Exp..

[16] Lee C., Shen C., Oubei H.M., Cantore M., Janjua B., Ng T.K., Farrell R.M., El-Desouki M.M., Speck J.S., Nakamura S. (2015). 2 Gbit/s data transmission from an unfiltered laser-based phosphor-converted white lighting communication system. Opt. Exp..

[17] Wei L.Y., Chow C.W., Chen G.H., Liu Y., Yeh C.H., Hsu C.W. (2019). Tricolor visible-light laser diodes based visible light communication operated at 40.665 Gbit/s and 2 m free-space transmission. Opt. Express.

[18] Lu H.H., Li C.Y., Lin H.H., Tsai W.S., Chu C.A., Chen B.R., Wu C.J. (2016). An 8 m/9.6 Gbps underwater wireless optical communication system. IEEE Photon. J..

[19] Huang X.H., Lu H.H., Chang P.S., Liu C.X., Lin Y.Y., Ko T., Chen Y.T. (2021). Bidirectional white-lighting WDM VLC–UWOC converged systems. J. Lightwave Technol..

[20] Armstrong J., Sekercioglu Y.A., Neild A. (2013). Visible light positioning: A roadmap for international standardization. IEEE Commun. Mag..

[21] Yang H., Zhong W.D., Chen C., Alphones A., Du P. (2020). QoS-driven optimized design-based integrated visible light communication and positioning for indoor IoT networks. IEEE Internet Things J..

[22] Palitharathna K.W.S., Wickramasinghe N.D., Vegni A.M., Suraweera H.A. (2024). Neural Network-Based Optimization for SLIPT-Enabled Indoor VLC Systems With Energy Constraints. IEEE Trans. Green Comm. Netw..

[23] Xie C., Guan W., Wu Y., Fang L., Cai Y. (2018). The LED-ID detection and recognition method based on visible light positioning using proximity method. IEEE Photon. J..

[24] Wang T.Q., Sekercioglu Y.A., Neild A., Armstrong J. (2013). Position accuracy of time-of-arrival based ranging using visible light with application in indoor localization systems. J. Lightw. Technol..

[25] Du P.F., Zhang S., Chen C., Alphones A., Zhong W.D. (2018). Demonstration of a low-complexity indoor visible light positioning system using an enhanced TDOA scheme. IEEE Photon. J..

[26] Hong C.Y., Wu Y.C., Liu Y., Chow C.W., Yeh C.H., Hsu K.L., Lin D.C., Liao X.L., Lin K.H., Chen Y.Y. (2020). Angle-of-arrival (AOA) visible light positioning (VLIP) system using solar cells with third-order regression and ridge regression algorithms. IEEE Photon. J..

[27] Hsu L.S., Chow C.W., Liu Y., Chang Y.H., Tsai D.C., Hung T.Y., Lin Y.Z., Jian Y.H., Yeh C.H. (2022). Utilizing single light-emitting-diode (LED) lamp and silicon solar-cells visible light positioning (VLP) based on angle-of-arrival (AOA) and long-short-term-memory-neural-network (LSTMNN). Opt. Comm..

[28] Kim H.S., Kim D.R., Yang S.H., Son Y.H., Han S.K. (2013). An indoor visible light communication positioning system using a RF carrier allocation technique. J. Lightw. Technol..

[29] Hsu C.W., Wu J.T., Wang H.Y., Chow C.W., Lee C.H., Chu M.T., Yeh C.H. (2016). Visible light positioning and lighting based on identity positioning and RF carrier allocation technique using a solar cell receiver. IEEE Photon. J..

[30] Hsu C.W., Liu S., Lu F., Chow C.W., Yeh C.H., Chang G.K. Accurate indoor visible light positioning system utilizing machine learning technique with height tolerance. Proceedings of the 2018 Optical Fiber Communications Conference and Exposition (OFC).

[31] Chuang Y.C., Li Z.Q., Hsu C.W., Liu Y., Chow C.W. (2019). Visible light communication and positioning using positioning cells and machine learning algorithms. Opt. Exp..

[32] Wu Y.C., Hsu K.L., Liu Y., Hong C.Y., Chow C.W., Yeh C.H., Liao X.L., Lin K.H., Chen Y.Y. (2020). Using linear interpolation to reduce the training samples for regression based visible light positioning system. IEEE Photonics J..

[33] Wu Y.C., Chow C.W., Liu Y., Lin Y.S., Hong C.Y., Lin D.C., Song S.H., Yeh C.H. (2020). Received-signal-strength (RSS) based 3D visible-light-positioning (VLP) system using kernel ridge regression machine learning algorithm with sigmoid function data preprocessing method. IEEE Access.

[34] Zhang S., Du P., Chen C., Zhong W.D., Alphones A. (2019). Robust 3D indoor VLP system based on ANN using hybrid RSS/PDOA. IEEE Access.

[35] Hsu L.S., Tsai D.C., Chow C.W., Liu Y., Chang Y.H., Lin Y.Z., Yeh C.H., Wang Y.C., Chen Y.Y. (2022). Using data pre-processing and convolutional neural network (CNN) to mitigate light deficient regions in visible light positioning (VLP) systems. J. Lightw. Technol..

[36] Fang S.H., Lin T. (2012). Principal component localization in indoor WLAN environments. IEEE Trans. Mob. Comput..

[37] Salamah A.H., Tamazin M., Sharkas M.A., Khedr M., Mahmoud M. (2019). Comprehensive investigation on principle component large-scale Wi-Fi indoor localization. Sensors.

[38] Dai J., Wang M., Wu B., Shen J., Wang X. (2023). A Survey of Latest Wi-Fi Assisted Indoor Positioning on Different Principles. Sensors.

[39] Chan H.M., Chow C.W., Liu Y., Yeh C.H., Chang Y.H., Hsu L.S., Tsai D.C., Yu T.W., Jian Y. (2022). H Using lighting design tool to simplify the visible light positioning plan and reduce the deep learning loading. Opt. Express.

